# Supplementary material for: Transcriptional landscape of PTEN loss in primary prostate cancer
Source: BMC Cancer. 2021 Jul 26;21:856. doi: 10.1186/s12885-021-08593-y (PMC8314517; doi:10.1186/s12885-021-08593-y)
Supplement: Supplementary file 2 — Additional file 2: Supplementary Figures. PDF file containing the raw results of all bioinformatics analysis. Figure S1. Cross-study of differential gene expression in PTEN-null vs PTEN-intact in ERG+ samples. Meta-analysis of HPFS/PHS and NH cohorts with Bayesian Hierarchical Model for DGE using XDE showing the top 25 most concordant differentially up- and down-regulated genes. PTEN status were based on IHC assays. Figure S2. PTEN expression levels stratified by CNV. Figure shows PTEN expression levels distribution by copy number variation (CNV), called by GISTIC algorithm. Figure S3. Correspondence-at-the-top (CAT) plot between TCGA CNV-based calls and the Bayesian Hierarchical Model approach (BHM). Agreement of genes ranked by t-statistics (TCGA) and average Bayesian Effect Size (BHM). Lines represent agreement between tested cohorts for PTEN-intact vs PTEN-null. Black-to-light grey shades represent the decreasing probability of agreeing by chance based on the hypergeometric distribution, with intervals ranging from 0.999999 (light grey) to 0.95 (dark grey). Lines outside this range represent agreement in different cohorts with a higher agreement than expected by chance. Figure S4. Expression of AC009478.1 is shown to be highly specific to PRAD, BLCA, to a lesser extent in UECA and BRCA. Figure shows raw expression values of SchLAP1 and AC009478.1 across cancer types. Pearson correlations and p-values are shown in red. Figure S5. Expression of FANTOM-CAT lncRNAs genes (top) and close coding genes (bottom) stratified by PTEN status. Significances based on t-test between PTEN-null and PTEN-intact using log2 CPM + 1 value. Significance cutoffs: * 0.05; **≤0.01; ***≤0.0001. Figure S6. Person correlation gene CATG00000038715 and CYP4F2 across cancer types. CATG00000038715 and CYP4F2 expression are shown to be highly correlated in PCa. Moreover, CATG00000038715 expression is shown to be highly specific to PCa. With exception of leukemia cells, none of the other tumors [file 12885_2021_8593_MOESM2_ESM.pdf]

Supplementary Figures

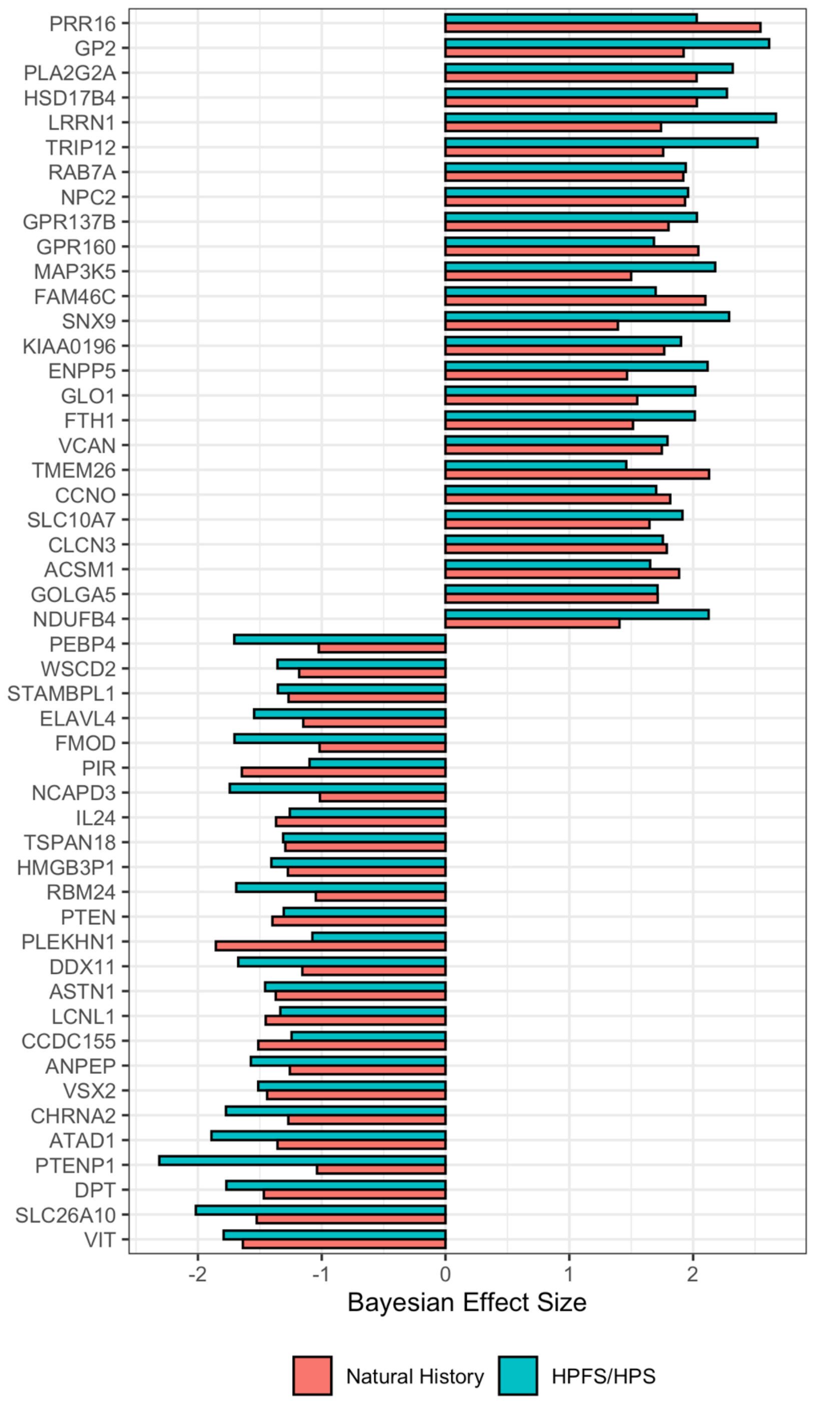

Figure S1. Cross-study of differential gene expression in PTEN-null vs PTEN-intact in ERG$^+$ samples. Meta-analysis of HPFS/PHS and NH cohorts with Bayesian Hierarchical Model for DGE using XDE showing the top 25 most concordant differentially up- and down-regulated genes. PTEN status were based on IHC assays.

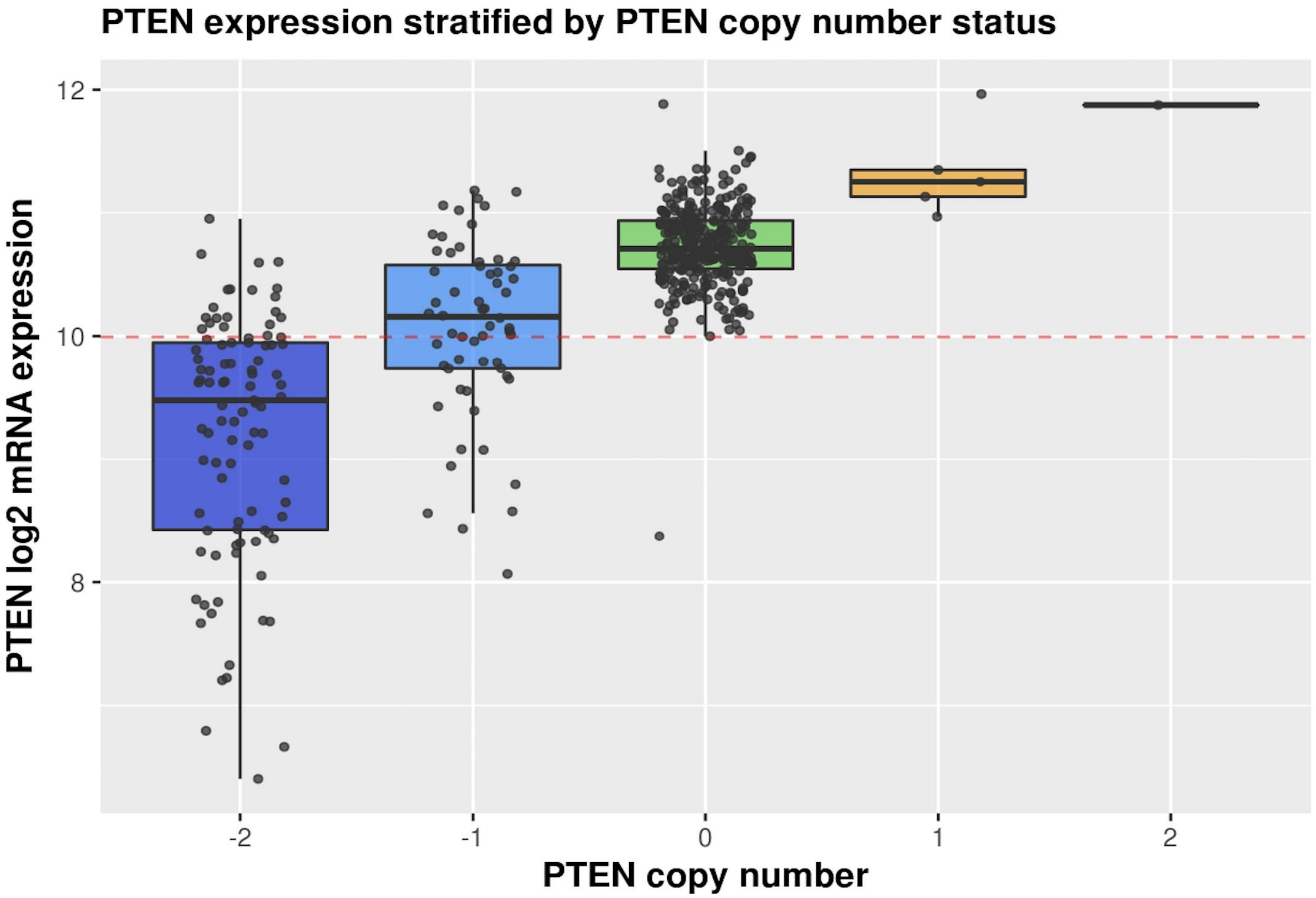

Figure S2. PTEN expression levels stratified by CNV. Figure shows PTEN expression levels distribution by copy number variation (CNV), called by GISTIC algorithm.

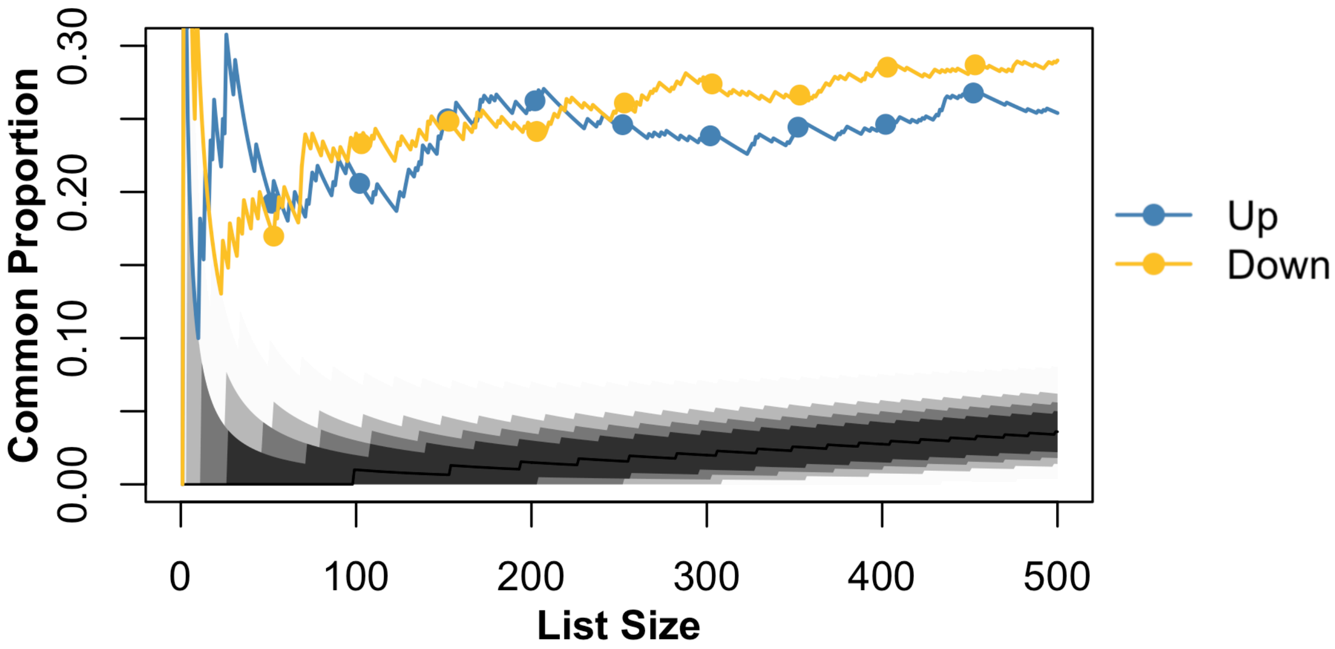

Figure S3. Correspondence-at-the-top (CAT) plot between TCGA CNV-based calls and the Bayesian Hierarchical Model approach (BHM). Agreement of genes ranked by t-statistics (TCGA) and average Bayesian Effect Size (BHM). Lines represent agreement between tested cohorts for PTEN-intact vs PTEN-null. Black-to-light grey shades represent the decreasing probability of agreeing by chance based on the hypergeometric distribution, with intervals ranging from 0.999999 (light grey) to 0.95 (dark grey). Lines outside this range represent agreement in different cohorts with a higher agreement than expected by chance.

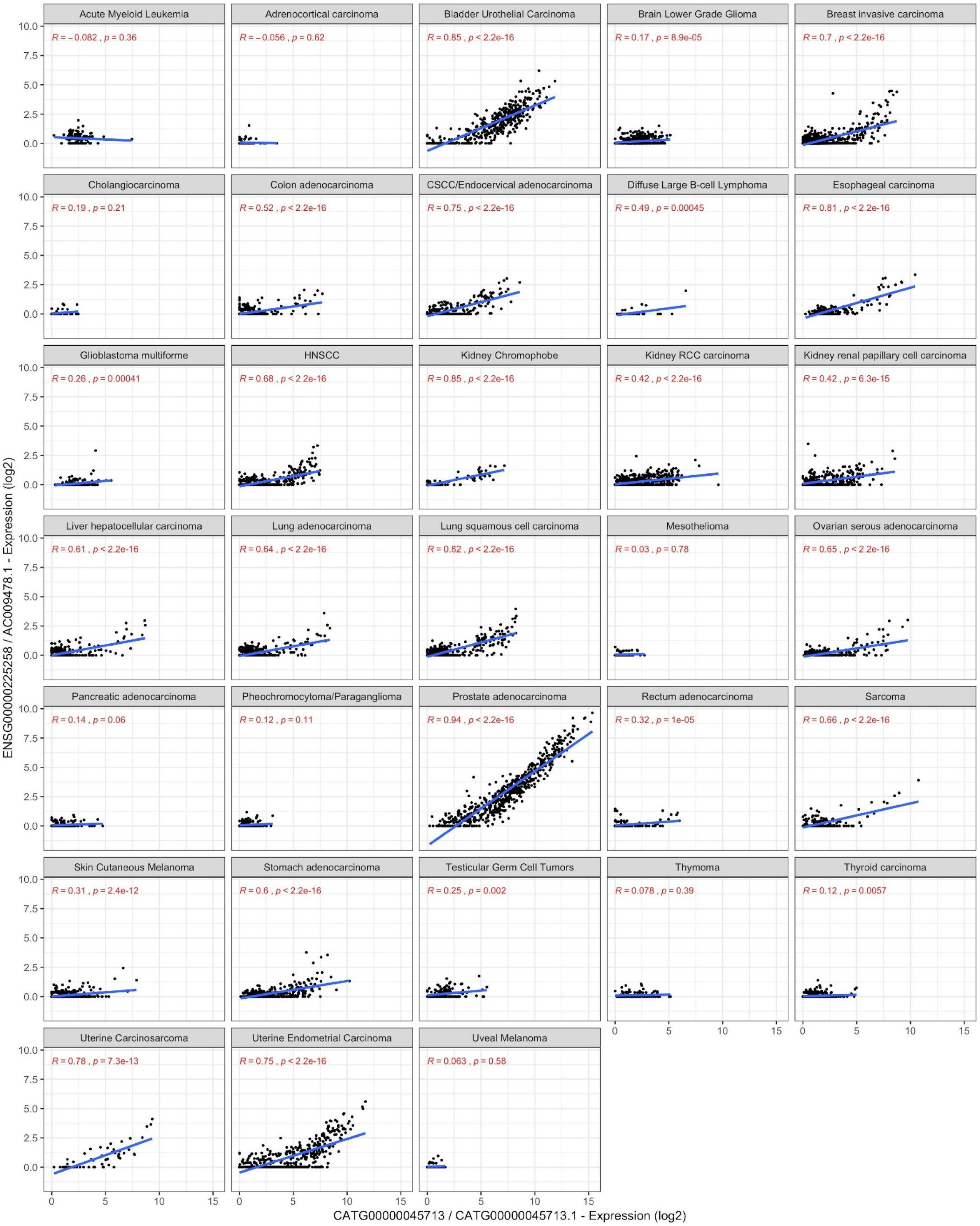

Figure S4. Expression of AC009478.1 is shown to be highly specific to PRAD, BLCA, to a lesser extent in UECA and BRCA. Figure shows raw expression values of SchLAP1 and AC009478.1 across cancer types. Pearson correlations and p-values are shown in red.

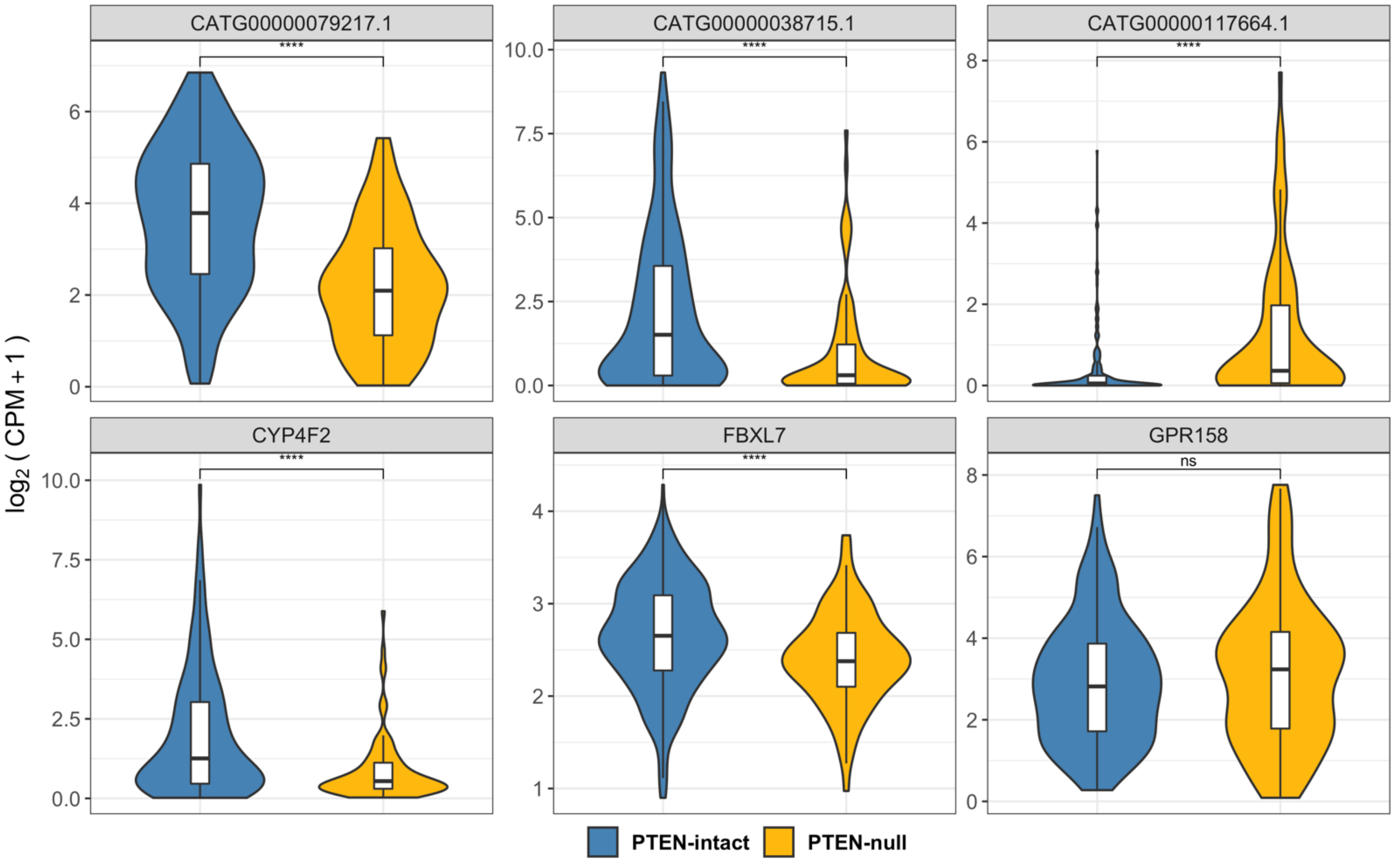

Figure S5. Expression of FANTOM-CAT lncRNAs genes (top) and close coding genes (bottom) stratified by PTEN status. Significances based on t-test between PTEN-null and PTEN-intact using $\log_2$ CPM+1 value. Significance cutoffs: *=≤ 0.05; **≤0.01; *** ≤ 0.001; ****≤0.0001.

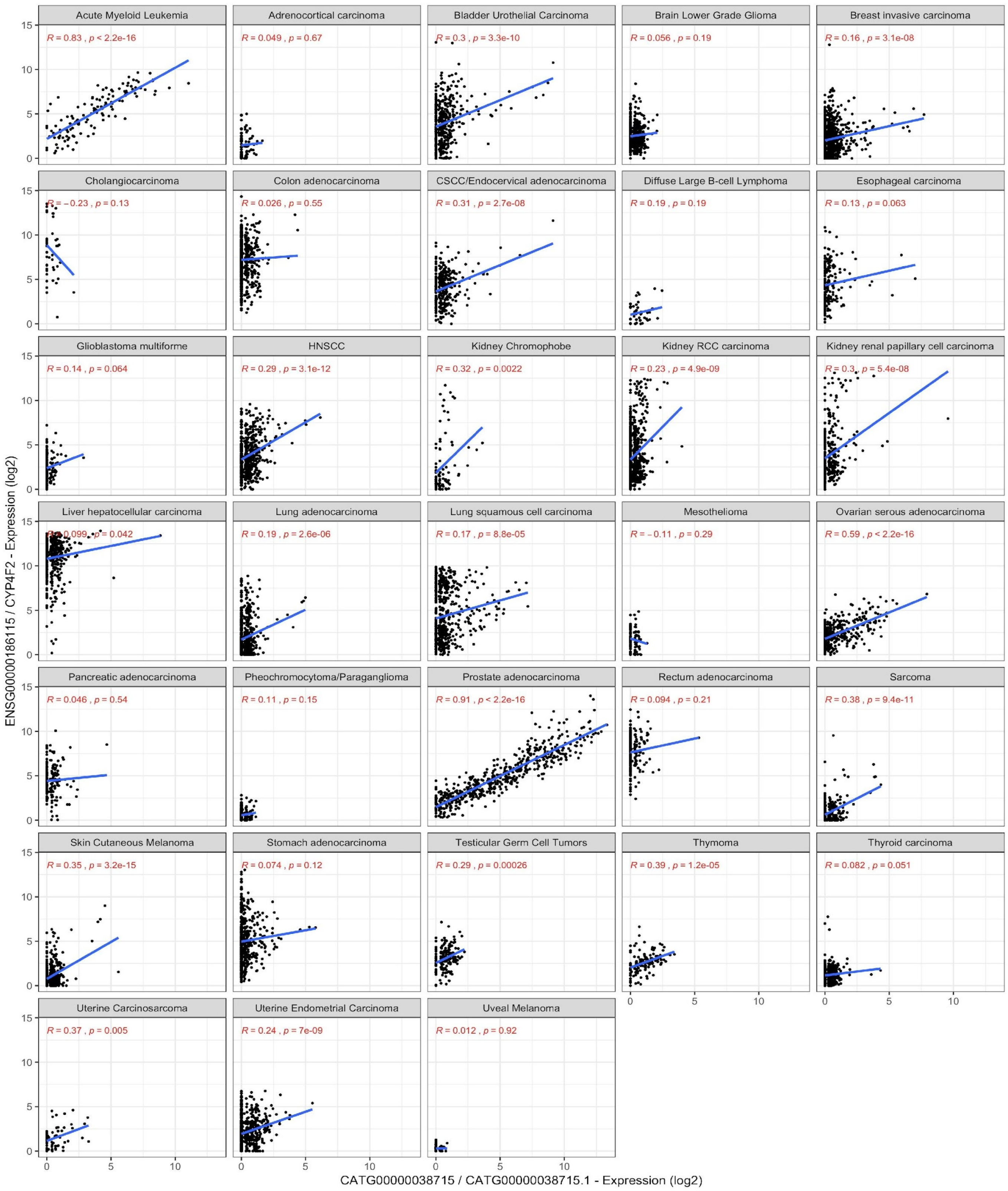

Figure S6. Person correlation gene CATG00000038715 and CYP4F2 across cancer types. CATG00000038715 and CYP4F2 expression are shown to be highly correlated in PCa. Moreover, CATG00000038715 expression is shown to be highly specific to PCa. With exception of leukemia cells, none of the other tumors expressed high levels of CATG00000038715.

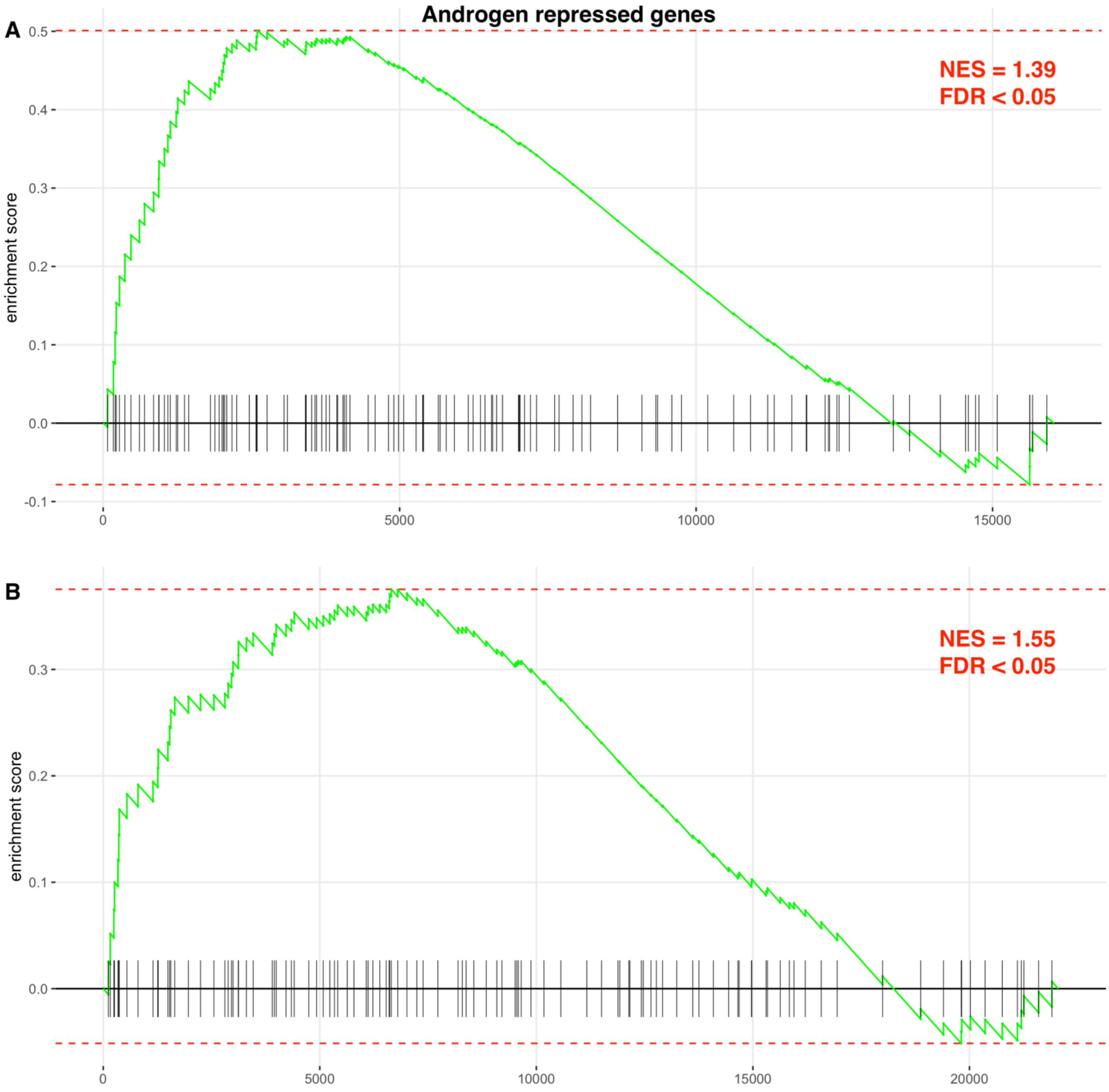

Figure S7. Gene set enrichment for Androgen repressed genes. Gene set enrichment analysis of gene signature showing positive enrichment of genes repressed by dihydrotestosterone after 6 hours of exposure obtained from Schaeffer et al.[48]. Enrichment for BHM-signature is shown in panel A and TCGA-signature in panel B.
